# Does free pregnancy testing reduce service denial in family planning clinics? A cluster-randomized experiment in Zambia and Ghana

**DOI:** 10.9745/GHSP-D-13-00011

**Published:** 2013-09-24

**Authors:** John Stanback, Gwyneth Vance, Gloria Asare, Prisca Kasonde, Beatrice Kafulubiti, Mario Chen, Barbara Janowitz

**Affiliations:** a FHI 360, Research Triangle Park, NC, USA; b Ghana Health Service, Accra, Ghana; c FHI 360/Zambia, Lusaka, Zambia; d Ministry of Health, Government of Zambia, Lusaka, Zambia

## Abstract

**Background:**

In many countries, pregnancy tests are not freely available in family planning clinics. As a result, providers sometimes deny services to non-menstruating clients due to uncertainty about pregnancy. Few clients are actually pregnant, yet denied clients run the risk of becoming pregnant, and those sent to pharmacies pay inflated prices for inexpensive tests. To assess the programmatic effect of free pregnancy testing, we conducted cluster-randomized trials in Ghana and Zambia, assessing clients' uptake of contraception in family planning clinics.

**Methods:**

In each country, 5 clinics were randomized to intervention status and 5 to control. Service data from 2,028 new, non-menstruating clients in Zambia and 1,556 in Ghana were collected. Intervention clinics received supplies of pregnancy tests, and staff were instructed to use tests as needed to help exclude pregnancy. Control clinics received no intervention. The primary outcome was the proportion of non-menstruating clients denied an effective contraceptive method. Cost-effectiveness was also evaluated.

**Results:**

In Zambia, clients in intervention and control clinics faced a similar risk of service denial at baseline, 15% and 17%, respectively. At follow-up, denial remained unchanged at 17% in control clinics, but decreased significantly to 4% in intervention sites. Clients in Zambia were 4.4 (95% confidence interval [CI] = 1.3–14.4) times more likely to be denied a method in control sites versus intervention sites (*P*<.01). Results from Ghana were inconclusive. Cost of a “denial averted” in Zambia was estimated to be US$0.59.

**Interpretation:**

Zambia results suggest that availability of free pregnancy testing significantly reduced contraceptive service denial, although results from Ghana preclude an unqualified recommendation. Authors conclude that free pregnancy testing in family planning clinics may make strong public health sense in those developing countries where denial to non-menstruating clients remains a problem. Although pregnancy can usually be excluded with a client history, pregnancy tests are often necessary.

## Abstract (precis)

Pregnancy tests, which cost very little (∼US$0.10) and are often required for successful family planning service delivery, may reduce service denial, and should be available in all family planning clinics at no or minimal cost to clients.

## BACKGROUND

Family planning providers in many developing countries still deny services to some non-menstruating clients due to uncertainty about pregnancy status.^1^^,^^2^ Such clients are sent away, either home to await menses or forced to go to a pharmacy to buy a pregnancy test. Resources are wasted as clients—very few of whom are pregnant^3^^,^^4^—pay for expensive, often unnecessary tests and make multiple clinic visits. Clients sent home to await menses also run the risk of becoming pregnant in the interim. The scope of this problem is unknown, and there is some evidence it may be decreasing. Older studies in Kenya suggested that nearly half of non-menstruating clients were denied immediate services,^5^ while more recent research has suggested proportions ranging from 17% to 35%.^6^ Still, the pool of those at risk is undoubtedly large; in many countries, nearly half of new family planning clients present for services during postpartum amenorrhea or between menstrual periods.^6^

Resources are wasted as non-menstruating clients—few of whom are pregnant—are denied family planning services, required to pay for expensive tests, or make multiple clinic visits.

## Checklists Not a Cure-All

**Figure 1.**
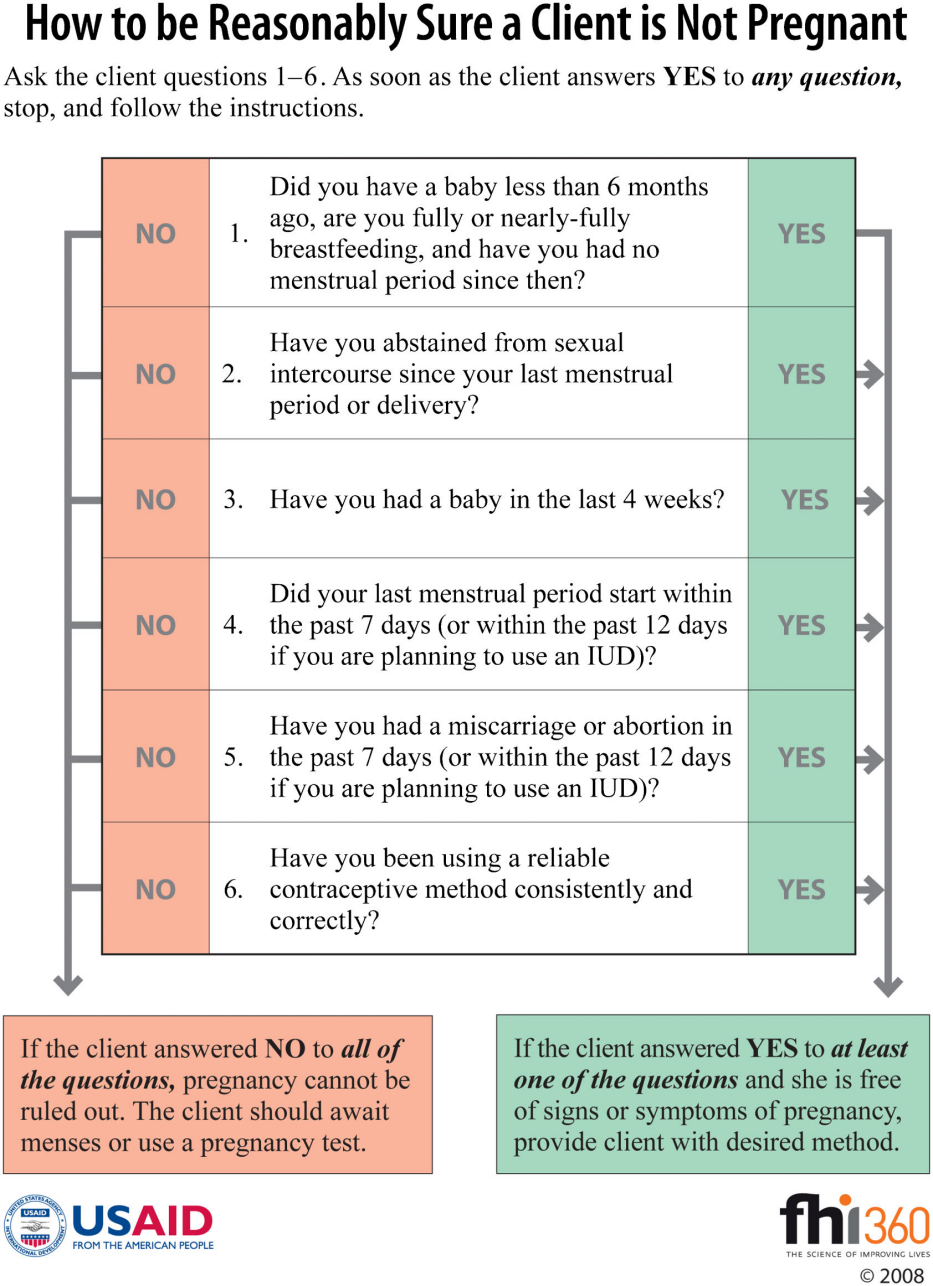
Pregnancy Checklist

In response to this problem, a job aid called the “pregnancy checklist” was developed to help family planning providers exclude pregnancy with a reasonable degree of certainty (Figure 1).^3^ Although this job aid—essentially a client history-taking tool—has been shown to improve access to services when used correctly,^6^ its introduction has not been a cure-all. Even when providers do take full advantage of the pregnancy checklist, it cannot exclude pregnancy for a sizable minority of new family planning clients who do not meet any one of its 6 criteria—such as sexual abstinence since last menstrual period—that rule out pregnancy.

## The Use of Pregnancy Tests

In wealthy countries, pregnancy tests are commonly used in family planning clinics and supplement, or substitute for, the client history on which the pregnancy checklist is based. In poorer countries, pregnancy tests may be perceived as too expensive for routine use for family planning. When health centers do stock limited numbers of test kits, our experience is that they are often reserved for antenatal care, although, in some countries, even those clients are forced to purchase tests from retail outlets.^7^

However, the price of highly accurate pregnancy tests has decreased significantly in recent years, and programs can now purchase simple paper strip tests for less than US$0.10,^8^ a price which may make free clinic-based testing cost-effective. Yet in spite of the low price of tests and the fact that the pregnancy checklist is ineffective in some situations, few poor countries have made free or inexpensive pregnancy testing a routine adjunct to family planning services, and the impact of providing free tests is unknown. To assess the programmatic effect of free pregnancy testing, we conducted cluster-randomized trials in Ghana and Zambia, assessing clients' immediate uptake of effective contraceptive methods in government family planning clinics. A cluster design was used because it was impracticable to randomize clients individually.

A cluster-randomized experiment assessed the programmatic effect of free pregnancy testing in family planning clinics in rural areas of Ghana and Zambia.

## METHODS

### Study Design and Population

The experiment was conducted in 2009–2010 as a sub-study “piggy-backed” onto a larger study being conducted in the Central Region of Ghana and in Central Province, Zambia. Both areas are largely rural, and are marked by poverty, high fertility, and low use of modern contraceptives. Before study initiation, the protocol received ethical approval from the Ghana Health Service Ethical Review Committee, the ERES Converge Institutional Review Board (IRB) in Zambia, and the Protection of Human Subjects Committee of FHI 360 in North Carolina, USA.

We hypothesized that free availability of pregnancy testing would reduce service denial rates in government family planning clinics. To calculate the required sample size, we used a z-approximation for comparisons of change from baseline between the groups using a logistic model with adjustments for clustering effects.^9^ Sample size calculations were based on estimates of the proportion of women denied effective contraceptive methods, the expected change in this level, as well the intraclass correlation coefficient (ICC)—a measure that quantifies the extent to which services to clients *within* a clinic are more similar to one another than services between different clinics—among study clusters (facilities) and time points. The expected change in denial was based on findings from a previous study^6^ in which the pregnancy checklist job aid was introduced in family planning clinics to reduce the proportion of women leaving without a method. We assumed a decrease in denial of 14% (for example, from 16% to 2%) in the intervention group beyond any changes in the control group. The ICC among family planning clinics in this same study for the stated outcome was low, ranging from 0.018 to 0.056 for the different countries in the study. For computations we assumed an ICC of 5%. The correlation pretest-posttest was assumed to be no smaller than 1.25%.

### Baseline Data Collection

With these assumptions, contraceptive uptake information was needed in each country from a minimum of 100 new and re-starting family planning clients in each of 10 clinics to have a power of 83% to detect a significant difference between the arms with a one-sided test (that is, greater decrease in the intervention group) at a 5% significance level. For this paper, analysis was limited to non-menstruating women, so the effective sample size and power would be lower under the same assumptions.

In both Ghana and Zambia, Ministry of Health officials provided a list of representative health centers in target districts. Microsoft Excel's random number generator was used to allocate 5 clinics from each country's list to receive an ample supply of free pregnancy tests and 5 clinics to serve as controls. Prior to making the tests available in the intervention clinics, baseline data were collected for approximately 3 months in all clinics from new and restarting clients seeking family planning services. During data collection, family planning providers used a simple log to anonymously record the following information about each client: date of service, method received, menstrual status, how pregnancy was excluded, and, if no method was received, the reason why not. Client menstrual status was recorded as currently menstruating, postpartum amenorrhea, or intermenstrual (between two normal menstrual periods).

### Intervention Phase

After baseline data collection was completed, the intervention phase of the study began. Providers in the clinics randomly allocated to receive pregnancy tests were shown how to properly use the tests and instructed to use them as needed to help rule out pregnancy among their family planning clients. Control clinics received no specific instructions about ruling out pregnancy among their family planning clients. Using a client history to exclude pregnancy is a recommended practice in both countries, and providers may have been exposed in the past to the pregnancy checklist. (The job aid was seen in some of the study clinics.) However, we purposefully did not include the checklist in the intervention or instruct providers in how to use pregnancy tests in conjunction with the pregnancy checklist, preferring rather to isolate the effect of the availability of free pregnancy testing when compared with standard care. We did not measure the checklist's use beyond asking providers to note in the log what means were used to rule out pregnancy.

Immediately after the pregnancy tests were provided to the intervention clinics, 3 months of follow-up data collection began in all 10 clinics in each country, using the same data collection log sheets. To analyze the data, SAS 9.2 was used to compare changes from baseline between the 2 study groups. A logistic mixed model was used to account for clustering at the facility level, and intermenstrual status was included as a covariate. Intervention effects, therefore, were evaluated using a comparison of odds ratios for changes between pre- and post-test between the 2 groups as estimated with the model. A separate model was fitted for each country.

The number of tests used in each clinic was tracked to produce an estimate of the incremental cost of reducing service denial, in other words, the cost per denial averted.

## RESULTS

### Client Characteristics

Overall, 44% of new clients (similar proportions in each country) were not menstruating when they presented for services (Table). These women are at particular risk of service denial due to uncertainty about their pregnancy status. Among the non-menstruating clients, those in Zambia were equally divided between those with postpartum/lactational amenorrhea and those between menstrual periods. In Ghana, nearly two-thirds (63%) of non-menstruating clients were amenorrheic. (The pretest sample size in Ghana was lower than anticipated due to a national strike during this period.) Although data quality was generally very good, client-specific data on *how* providers excluded pregnancy were incomplete and thus were not included in this analysis.

**Table.** Family Planning Client Menstrual Status, by Study Group

	**Intervention Group**		**Control Group**	
	**(free pregnancy tests)**		**(standard care)**	
**Zambia**	**Pre (n = 439)**	**Post (n = 362)**	**Pre (n = 693)**	**Post (n = 534)**
Menstruating	52%	54%	42%	58%
Between menses	19%	19%	36%	26%
Amenorrheic	29%	27%	22%	16%
**Ghana**	**Pre (n = 239)**	**Post (n = 423)**	**Pre (n = 283)**	**Post (n = 611)**
Menstruating	53%	47%	46%	48%
Between menses	31%	20%	24%	15%
Amenorrheic	16%	33%	30%	37%

### Service Denial Outcomes

**Figure 2.**
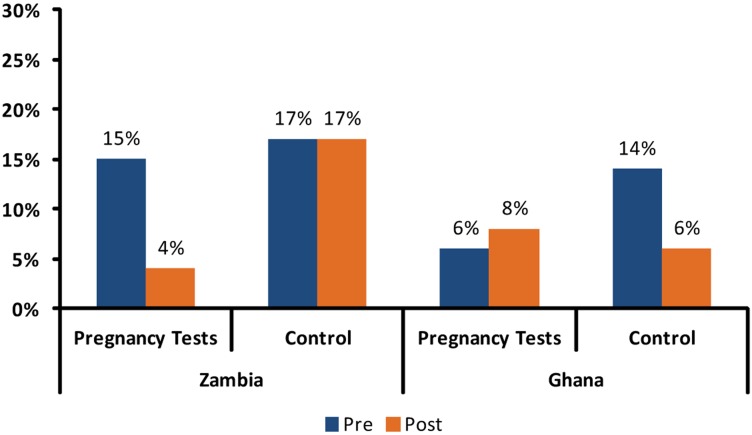
Denial of Contraceptive to Non-Menstruating Family Planning Clients

The primary outcome assessed was the denial of an effective contraceptive method to non-menstruating family planning clients (Figure 2). In Zambia, clients in both intervention and control clinics faced a similar risk of service denial at baseline, 15% and 17% respectively. At follow-up, service denial remained unchanged at 17% in the five control clinics, but decreased significantly to only 4% in intervention sites. Our model (not shown), which accounted for clustering at the clinic level and adjusted for intermenstrual status, estimated that clients in Zambia were 4.4 (95% confidence interval [CI] = 1.3–14.4) times more likely to be denied a family planning method in control sites as compared with intervention sites where free pregnancy tests had been introduced, with a one-sided *P* value of <.01. This comparison accounts for baseline levels.

Clients in Zambia were 4.4 times more likely to be denied a family planning method in control sites as compared with intervention sites where free pregnancy tests had been introduced.

The results from Ghana were less clear. At baseline, risk of denial was dissimilar in the 2 clinic groups (6% in intervention sites and 14% in controls). Subsequently, denial rates remained stable in intervention clinics (8%), but decreased to 6% in *control* clinics (*P* = .85).

### Cost per “Denial Averted”

Providers used 117 pregnancy tests in the intervention clinics in Zambia and 200 tests in Ghana. We divided the total costs of tests used (US$0.09 per test) in the post-intervention period by the estimated number of clients who, in the absence of free pregnancy testing, would have been denied services. Based on the differences in the pre- and post-intervention levels of denials, we calculated that, in Zambia, 18 clients would have been denied services but were not, due to the availability of testing. Given the costs of tests used during that period, the estimated cost of a “denial averted” in Zambia was only US$0.59. Although the intervention did not appear to prevent denials in Ghana, we pooled the cost of tests from both countries and found that, study-wide, the estimated cost of a denial averted was US$1.59.

## DISCUSSION

### Reduced Service Denial

Taken alone, the results from Zambia suggest that the availability of free pregnancy testing significantly reduced contraceptive service denial in government clinics in that country. After discussions with the Ghana country research team, we still have no full explanation for the results in Ghana, in which no change was seen in the intervention sites, but denial rates appeared to have dropped in *control* clinics during the time of the study. We feel that the most likely explanation is that service denial in Ghana is low, on the order of 6%–8%, and cannot, therefore, decrease substantially. We speculate that the relatively high level of denial (14%) seen in the control group during the pre-test phase was an aberration, perhaps related to the national health workers' strike occurring at that time, which simply reverted to a more normal level during the post-test period.

### A Public Health Rationale

While the inconclusive results from Ghana preclude an unqualified recommendation, results from Zambia suggest that availability of free or low-priced pregnancy tests in family planning clinics may make strong public health sense in developing countries where service denial to non-menstruating clients remains a problem. Pregnancy test strips cost very little and fill an obvious gap when a client history fails to exclude pregnancy. When testing is indicated (for example, when the pregnancy checklist fails to rule out pregnancy in a woman with amenorrhea), women in such settings may be unable to afford these products from private pharmacies, where similar tests retail for between US$1 and $3 (a mark-up of greater than 1,000%). In rural areas, pharmacies and drug retailers that sell pregnancy tests are often nonexistent.

The availability of free or low-priced pregnancy tests may make strong public health sense where service denial to non-menstruating clients remains a problem.

### Low Costs

What prevents clinics from offering free or inexpensive pregnancy testing now? Currently, programs in the poorest developing countries do not usually include pregnancy tests in the “basket” of contraceptive commodities provided to clinics, and adding a new product is as much a logistic headache as a financial one. Of course, commodity costs are only part of the final cost of providing such a product at scale, and yet given the very low cost of pregnancy tests—in this study, the commodity cost for 3 months' worth of tests for 5 clinics was only about US$10—and their potential role in improving women's access to family planning, a strong case can be made for routine procurement of tests similar to that of other complementary products, such as latex gloves and sharps containers.

### A Compatible Tool

If pregnancy tests do become more widely available in family planning programs in developing countries, a strong role should remain for the simple, low-tech pregnancy checklist to help providers rule out pregnancy. Neither histories nor pregnancy tests are perfect screening tools; “false negatives” can occur when pregnancy tests miss early pregnancies, and, more commonly, “false positives” occur (with published frequency ranging from 12%^3^ to 39%^4^) when the pregnancy checklist fails to exclude pregnancy in non-pregnant women.^1^ But since no known risk occurs to either a mother or an undetected fetus from exposure to contraceptive hormones, the benefits of increased client contraceptive access should take precedence over excessive caution in excluding pregnancy, particularly in countries with high maternal mortality and morbidity rates. Every effort should be made to provide effective contraception on the same day that a woman presents for contraceptive services.

Every effort should be made to provide effective contraception on the same day that a woman presents for contraceptive services.

### Added Benefits

Wider availability of pregnancy tests could have other benefits as well. Free or nominally priced testing could encourage women to enter the health system to know their pregnancy status. If pregnant, they could get a timely referral for antenatal care^7^ or, where legal and if the women desire, pregnancy termination. For non-pregnant women, testing could serve as a “teachable moment” to offer family planning services. Pregnancy tests are also useful to exclude pregnancy in the common situation when women present more than one month late for DMPA re-injection^10^ or to provide reassurance in cases where women using progestin-only methods, such as contraceptive implants, worry that they are pregnant when they experience the normal side effect of amenorrhea.

### Training Remains Important

Family planning providers in developing countries should be trained to know when to use a client history or a pregnancy test—or both—to exclude pregnancy. The 2 tools are complementary and should be used in a way that optimizes successful service delivery. For example, if a woman is amenorrheic (such as during the postpartum period or after discontinuation of progestin-only injectable), the preferable hierarchy is that the pregnancy checklist should be used first, since tests, although cheap, are not free. If the checklist fails to rule out pregnancy, then a pregnancy test should be used. On the other hand, if a woman presents between 2 normal menstrual periods, a pregnancy test is not useful. If a woman has already missed her menstrual period, a pregnancy test should be used to rule out or confirm pregnancy. In retrospect, we realize that one limitation of the study was that the design used did not better reflect the complementarity between pregnancy tests and the pregnancy checklist. Ideally, a 3-arm or factorial design should have been used.

The pregnancy test and the checklist are complementary tools and should be used in a way that optimizes successful service delivery.

### Next Steps

If family planning programs decide to incorporate free pregnancy testing, a key next step would be to address procurement considerations, such as supply chain logistics, so that pregnancy tests are reliably delivered to clinics along with other supplies. Meanwhile, training for family planning providers should include appropriate use of both methods to exclude pregnancy, emphasizing the provider-client trust required for appropriate use of the pregnancy checklist. Additional research on reasons for service denial by providers would also be useful, given that entities such as the U.S. Agency for International Development (USAID) and the World Health Organization have been working for years to eliminate this medical barrier.

## References

[1] Campbell M Sahin-Hodoglugil NN Potts M Barriers to fertility regulation: a review of the literature. Stud Fam Plann. 2006;37(2): 87–98 10.1111/j.1728-4465.2006.00088.x 16832983

[2] Stanback J Thompson A Hardee K Janowitz B Menstruation requirements: a significant barrier to contraceptive access in developing countries. Stud Fam Plann. 1997;28(3): 245–250 10.2307/2137892 9322340

[3] Stanback J Qureshi Z Sekadde-Kigondu C Gonzalez B Nutley T Checklist for ruling out pregnancy among family-planning clients in primary care. Lancet. 1999;354(9178): 566 10.1016/S0140-6736(99)01578-0 10470704

[4] Stanback J Nanda K Ramirez Y Rountree W Cameron SB Validation of a job aid to rule out pregnancy among family planning clients in Nicaragua. Rev Panam Salud Publica. 2008;23(2): 116–118 10.1590/S1020-49892008000200007 18371282

[5] Stanback J Griffey S Lynam P Ruto C Cummings S Improving adherence to family planning guidelines in Kenya: an experiment. Int J Qual Health Care. 2007;19(2): 68–73 10.1093/intqhc/mzl072 Medline 17277011

[6] Stanback J Diabate F Dieng T Duarte de Morales T Cummings S Traoré M Ruling out pregnancy among family planning clients: the impact of a checklist in three countries. Stud Fam Plann. 2005;36(4): 311–315 10.1111/j.1728-4465.2005.00073.x 16395948

[7] Morroni CA Moodley J The role of urine pregnancy testing in facilitating access to antenatal care and abortion services in South Africa: a cross-sectional study. BMC Pregnancy Childbirth. 2006;6(1): 26 10.1186/1471-2393-6-26 16893459 PMC1555610

[8] Management Sciences for Health. International drug price indicator guide. Cambridge, MA: Management Sciences for Health, 2012 Available from: http://erc.msh.org/mainpage.cfm?file = 1.0.htm&module = DMP&language = english

[9] Preisser JS Reboussin BA Song EY Wolfson M The importance and role of intracluster correlations in planning cluster trials. Epidemiology. 2007;18(5): 552–560 10.1097/EDE.0b013e3181200199 17879427 PMC2567827

[10] Baumgartner JN Morroni C Mlobeli RD Otterness C Buga G Chen M Impact of a provider job aid intervention on injectable contraceptive continuation in South Africa. Stud Fam Plann. 2012;43(4): 305–314 10.1111/j.1728-4465.2012.00328.x 23239249

